# Pet-related *Pasteurella multocida* induced peritonitis in peritoneal dialysis: a case report and review of the literatures

**DOI:** 10.1186/s12882-020-01765-1

**Published:** 2020-03-19

**Authors:** Haoran Mu, Man Yang, Yueyue Zhang, Yajing Zhang, Juan Wang, Weijie Yuan, Shu Rong

**Affiliations:** 1 Shanghai Jiao Tong University School of Medicine, Shanghai, China; 2 Department of Nephrology, Shanghai General Hospital, Shanghai Jiao Tong University School of Medicine, Shanghai, 200080 China

**Keywords:** Peritoneal dialysis, Peritonitis, *Pasteurella multocida*, Pet, Cat

## Abstract

**Background:**

*P. multocida (Pasteurella multocida)* is animal-sourced gram-negative coccobacillus which can be transmitted to human through many animals including household pets. *P. multocida* induced peritoneal dialysis-related peritonitis has rarely been reported. In recent years, there has been an increase in the incidence of *P. multocida* induced peritoneal dialysis-related peritonitis, for the reason that patients with PD at home bred household pets. In this study, we present a case of a *P. multocida* induced peritoneal dialysis-related peritonitis, which is suspected to be caused through intimate contact with a household cat and we have reviewed 28 cases reported before and give suggestions for treatment and the way of prevention.

**Case presentation:**

A 75-year-old man with end-stage renal disease (ESRD) for nearly 5 years on continuous ambulatory peritoneal dialysis (CAPD) was admitted to the nephrology department with a 1-week history of abdominal pain and a cloudy peritoneal dialysis effluent. Based on the history, physical examination and laboratory results with the findings in the peritoneal dialysis fluid, a diagnosis of peritoneal dialysis-related peritonitis was confirmed. The final culture of initial peritoneal effluent results indicated the organism was *P. multocida.* After a 12-day antibiotic treatment, the condition of patient was not improved. *Th*e patient was switched to ampicillin/sulbactam (3 g intravenously) twice every day and the condition was improved significantly. On further inquiring, the patient reported that he had had a cat at home and when the patient did CAPD, the cat was usually playing with the tubing or contacting the patient during CAPD.

**Conclusion:**

In our case and reviewed cases, *P. multocida* induced peritoneal dialysis-related peritonitis could be cured by proper antibiotic treatment. If individuals keep the pet away from the PD process, the infection route may be severed. *P. multocida* induced peritoneal dialysis-related peritonitis does not need catheter removal and exchange with hemodialysis except long-time intractable peritonitis.

## Background

*P. multocida (Pasteurella multocida)* is animal-sourced gram-negative coccobacillus which can be transmitted to human through many animals including household pets, such as cats [1]. Commonly *P. multocida* has been reported to cause human soft tissue infection through being bitten by pets and *P. multocida* induced peritoneal dialysis-related peritonitis has rarely been reported. The first case of infection was reported by Paul and Rostand in 1987 and in recent years, there has been an increase in the incidence of *P. multocida* induced peritoneal dialysis-related peritonitis, for the reason that patients with PD at home bred household pets [2].

In this study, we present a case of a *P. multocida* induced peritoneal dialysis-related peritonitis, which is suspected to be caused through intimate contact with the household cat. Furthermore, we have reviewed 28 cases reported before and suggest more effective management on antibiotic treatment and the way of prevention.

## Case presentation

**Fig. 1:**
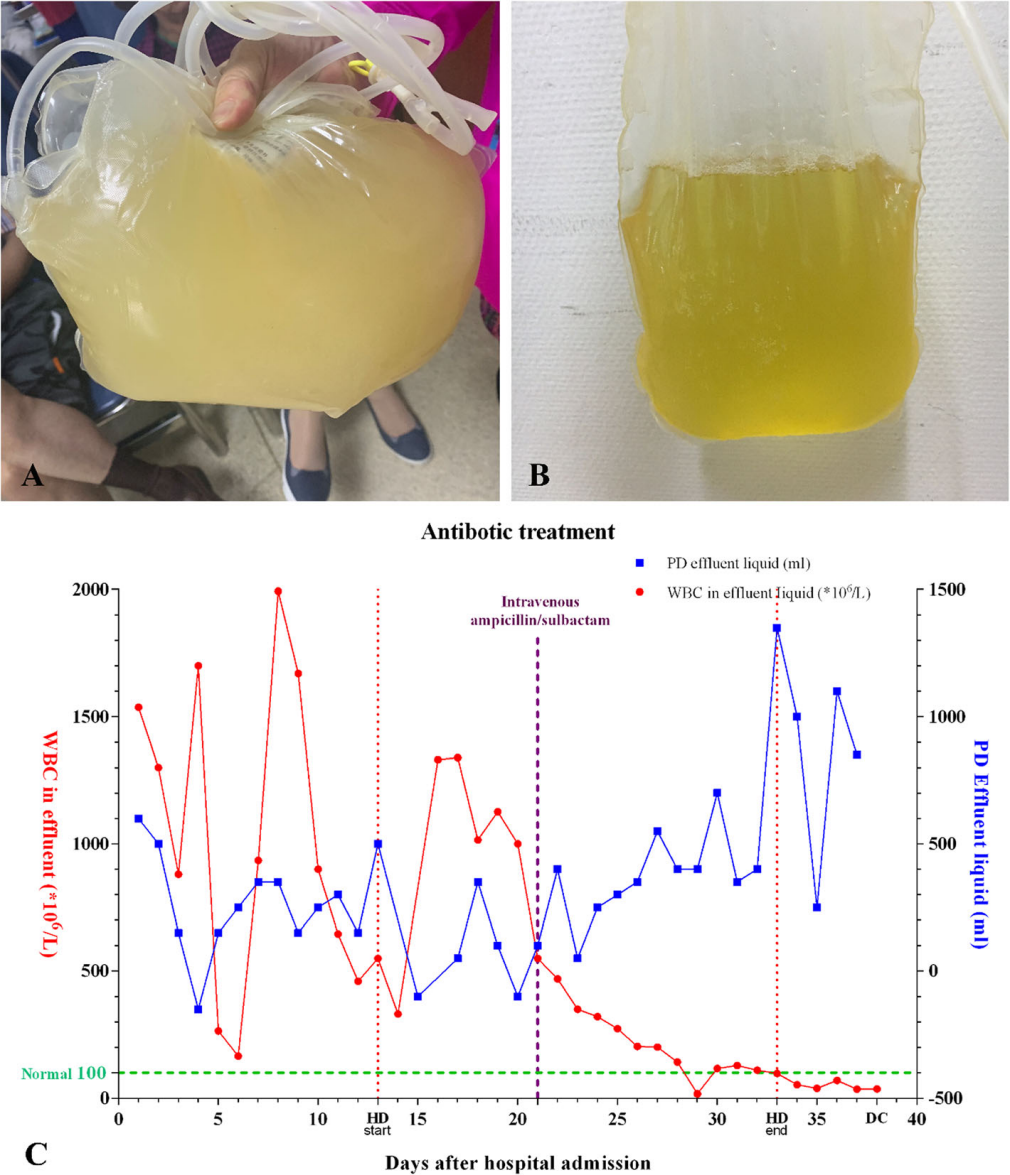
The character of PD effluent. **a** The effluent collected on the day of admission. **b** The effluent collected to compare on the day of discharge. **c** Antibiotic treatment after hospital admission. At the early stage of treatment, we tried different types of antibiotics according drug sensation test. However, the effectiveness was not satisfied. After using ampicillin/sulbactam intravenously on Aug. 30th, the WBC count in PD effluent went down and the volume of the effluent went up, indicating an effective treatment. During the treatment, the patient had a period of temporary hemodialysis from the 13th day after admission to the day that WBC count of PD effluent was normal stably after 12 days ampicillin/sulbactam treatment. HD: hemodialysis, DC: discharge

On Aug. 9th, 2019, a 75-year-old man with end-stage renal disease (ESRD) on continuous ambulatory peritoneal dialysis (CAPD) for 5 years, which was 1.5% low-calcium dialysate in a 4-to-5-h dwell three times every day and 2.5% low-calcium dialysate in an 8-to-10-h dwell once every night, was admitted to the nephrology department with an 1-week history of abdominal pain and a cloudy peritoneal dialysis effluent (Fig. 1a). The patient had a past medical history of hypertension. For the reason that his pressure was lower than before, the antihypertensive drugs were stopped on admission.

Physical examination revealed an emaciated man with a blood pressure of 110/75 mmHg, a breath of 16 beats/min, a pulse of 76 beats/min and a temperature of 36.8 °C. A head examination revealed no face edema. The cardiopulmonary exam was unremarkable. Abdominal examination revealed a soft abdomen, with moderate tenderness to palpation throughout particularly at the lower abdomen, moderate guarding with no rebound and normative bowel sounds. There was no discharge or erythema at the peritoneal dialysis catheter exit site. The tubing of the peritoneal dialysis catheter was intact and there was no macroscopic evidence of the peritoneal dialysis catheter damage.

Laboratory tests on the admitted day were available. The blood routine examination was as follows: white blood cell (WBC) count of 5.00 × 109/L, a neutrophil ratio of 78.2%, lymphocyte ratio of 14.1%, red blood cell (RBC) count of 3.43 × 1012/L, hemoglobin of 83.10 g/L and platelet count of 110 × 109/L. The biochemical routine examination was as follows: sodium 137.00 mmol/L, potassium 2.97 mmol/L, chloride 95.0 mmol/L, glucose 7.4 mmol/L, blood urea nitrogen 21.76 mmol/L and creatinine 878.2 μmol/L. BNP (natriuretic peptide B) was 135 pg/ml and myoglobin was 402.4 ng/ml. The initial peritoneal effluent analysis further revealed as follows: WBC count of 1537.0 × 106/L, chloride 105.8 mmol/L, glucose 20.8 mmol/L, total protein 2.59 g/L, lactic dehydrogenase (LDH) 54.8 U/L, adenosine deaminase (ADA) 0.9 U/L. The changes of important laboratory results were being detected during the admitted period (Fig. 1c).

Based on the history, physical examination and laboratory results with the findings in the peritoneal dialysis fluid, a diagnosis of peritoneal dialysis-related peritonitis was confirmed. Besides related symptomatic treatments, the empiric antibiotic treatment was initiated with levofloxacin (0.5 g intravenously) every day, ceftazidime (0.25 g intraperitoneally) in a 3-h dwell four times every day and vancomycin (1.0 g intraperitoneally) in an 8-h dwell once at night every 5 days. Final culture of initial peritoneal effluent results indicated the organism was *P. multocida,* which was found to be sensitive to ampicillin/sulbactam, cefazolin, cefotaxime, cefoxitin, levofloxacin, ampicillin, cefuroxime, imipenem, ciprofloxacin, ceftazidime, meropenem, and cefoperazone/sulbactam, while in the blood there were no bacterial infections were having been found. According to the drug-sensitive test, the intravenously antibiotic treatment was switched to meropenem (0.5 g intravenously) every 12 h, with peritoneal antibiotic treatment still. After one-day treatment, the symptoms disappeared but the WBC count of peritoneal effluent was still above the normal level of the WBC counts, which reminded the infection remained existed. Therefore, Cefazidime was switched to amikacin (200 mg intraperitoneally) in a 3-h dwell four times every day and on Aug. 18th, intravenously meropenem was switched to cefoperazone/sulbactam (1.5 g intravenously) every 12 h. And for the reason that the CAPD was noneffective and the peritoneal infection was not controlled, the patient was undergone temporary hemodialysis four times every week with imipenem/cilastatin (500 mg intraperitoneally) in a 6-h dwell every day for continuing peritoneal antibiotic treatment. After 12-day antibiotic treatment, the WBC count of peritoneal effluent was not significantly improved and the peritoneal effluent was still cloudy. When it was the deadline of the ISPD guideline recommending to hemodialysis, the patient firmly refused the lasting hemodialysis and asked for a further treatment. According to the drug-sensitive results and reviewed case reports (Table 1), the patient was switched to ampicillin/sulbactam (3 g intravenously) twice every day. The WBC count of peritoneal effluent was markedly improved and the antibiotic treatment was continued until the WBC count of the peritoneal effluent was below 100 × 106/L and the PD effluent was clear, which revealed the infection was controlled (Fig. 1b, c). During the treatment, the patient was undergone temporary hemodialysis 12 times to maintain the function of excretion. When the infection was controlled and the function of CAPD recovered, the temporary hemodialysis was stopped and the patient was returned to CAPD. The patient discharged after using ampicillin/sulbactam for 17 days and continued amoxicillin (0.25 g orally) three times a day for another 6 days. The detail key dates of altering antibiotics and methods have been listed (Table 2).

**Table 1:** Review of the cases in the previous literatures

Case	Reference	Sex	Age(Yr)	Main complains	PD effluent characters	Animal exposure	Effective treatments	Results
1	Rondon-Berrios, H. [3]	Male	38	Severe and diffuse abdominal pain	Cloudy	Household cat	Piperacillin/tazobactam (IV) Vancomycin (IV)	Hemodialysis
2	Campos, A. [4]	Male	8	Diffuse abdominal pain	Cloudy	Household hamster	Tobramycin (IP)	Peritoneal dialysis
3	Sol, P. M. [5]	Female	7	Abdominal pain and vomiting	Cloudy	Household cat	Ampicillin (IP)	Peritoneal dialysis
4	Paul, R. V. [2]	Female	55	Severe abdominal pain	Milk-colored	Household cat	Vancomycin (IV) gentamicin (IV)	Peritoneal dialysis
5	Cooke, F. J. [6]	Female	73	Abdominal pain	Cloudy	Household cat	Gentamicin (IP) Ciprofloxacin (PO)	Peritoneal dialysis
6	Dresselaars, H. F. [7]	Female	62	Mild abdominal discomfort	Turbid	Household cat	Cotrimoxazole (IV) Cefalotin (IP)	Peritoneal dialysis
7	Giron, F. F. [8]	Male	72	Abdominal pain	Turbid	Household cat	vancomycin (IP) ceftazidime (IP)	Peritoneal dialysis
8	Satomura, A. [9]	Male	58	Abdominal discomfort	Unknown	Household cat	cefazolin (IP) ceftazidime (IP)	Peritoneal dialysis
9	Joh, J. [10]	Male	55	Abdominal pain, nausea and vomiting	Cloudy	Household cat	Gentamicin (IP) Ampicillin/sulbactam (PO)	Peritoneal dialysis
10	Kim, I. [11]	Female	25	Diffuse abdominal pain	Cloudy	Household cat	Cefazolin (IP) Gentamicin (IP)	Peritoneal dialysis
11	Loghman-Adham, M. [12]	Female	12	Mild abdominal pain	Clear	Household cat	Cephapirin (IP) Gentamicin (IP)	Peritoneal dialysis
12	MacKay, K. [13]	Male	73	Mild abdominal discomfort	Cloudy	Household cat	Vancomycin (IP) Ceftazadime (IP)	Peritoneal dialysis
13	Nishina, M. [14]	Male	45	Abdominal pain	Cloudy	Household cat	Vancomycin (IV) Ceftazidime (IP)	Peritoneal dialysis
14	Freeman, A. F. [15]	Female	14	Abdominal pain	Cloudy	Household hamster	Vancomycin (IP) Ceftazadime (IP) Ampicillin/sulbactam (IV)	Peritoneal dialysis
15	Kanaan, N. [16]	Female	24	Diffuse abdominal pain and nausea	Turbid	Household cat	Vancomycin (IV) Ciprofloxacin (PO)	Peritoneal dialysis
16	Sillery, J. [17]	Female	48	General abdominal discomfort	Unknown	Household cat	Ampicillin (IV)	Peritoneal dialysis
17	Elsey, R. M. [18]	Male	25	Abdominal pain and nausea	Cloudy	Household cat	Cephradine (IP) Gentamicin (IP)	Peritoneal dialysis
18	London, R. D. [19]	Male	54	Abdominal pain, nausea and vomiting	Cloudy	Household cat	Vancomycin (IV) Gentamicin (IV)	Peritoneal dialysis
19	Mugambi, S. M. [20]	Female	36	Abdominal pain, nausea and vomiting	Cloudy	Household cat	Vancomycin (IV, IP) Gentamicin (IV, IP)	Peritoneal dialysis
20	Poliquin, P. G. [21]	Female	28	Severe abdominal pain	Cloudy	Household cat	Cefazolin (IP) Tobramycin (IP) Ceftazidime (IP)	Peritoneal dialysis
21	Poliquin, P. G. [21]	Male	37	Abdominal pain	Cloudy	Household cat	Cefazolin (IP) Tobramycin (IP)	Peritoneal dialysis
22	Poliquin, P. G. [21]	Male	41	Abdominal pain, nausea, vomiting and diarrhea	Cloudy	Household cat	Cefazolin (IP) Tobramycin (IP)	Peritoneal dialysis
23	Poliquin, P. G. [21]	Female	51	Abdominal pain, nausea and vomiting.	Cloudy	Household cat	Cefazolin (IP) Tobramycin (IP)	Peritoneal dialysis
24	Poliquin, P. G. [21]	Female	37	Abdominal pain, chills and diarrhea.	Cloudy	Household cat	Cefazolin (IP) Ceftazidime (IP)	Peritoneal dialysis
25	Poliquin, P. G. [21]	Female	59	Abdominal pain, nausea and vomiting	Unknown	Household cat	Cefazolin (IP) Tobramycin (IP)	Peritoneal dialysis
26	Poliquin, P. G. [21]	Female	69	Abdominal pain	Cloudy	Household cat	Cefazolin (IP) Tobramycin (IP)	Peritoneal dialysis
27	Van Langenhove, G. [22]	Female	22	Heavy abdominal pain	Cloudy	Household cat	Vancomycin (IP) Amikacin (IP) Ciprofloxacin (PO)	Peritoneal dialysis
28	Weiss, G. A. [23]	Male	57	Diffuse abdominal pain	Cloudy	Household cat	Vancomycin (IP) Ceftazadime (IP)	Peritoneal dialysis
29	This case	Male	75	Abdominal pain	Cloudy	Household cat	Ampicillin/sulbactam (IV)	Peritoneal dialysis

*IV* Intravenously, *IP* Intraperitoneal, *PO* per os

**Table 2:** Key altered dates of changing selected antibiotics and methods

Key Altered Dates	Selected Antibiotics		
	Orally	Intraperitoneally	Intravenously
2019/8/9	–	Vancomycin (1.0 g intraperitoneally) in an 8-h dwell once at night and ceftazidime (0.25 g intraperitoneally) in a 3-h dwell four times every day	Levofloxacin (0.5 g intravenously) every day
2019/8/15	–	Vancomycin (1.0 g intraperitoneally) in an 8-h dwell once at night	–
2019/8/18	–	–	Stop levofloxacin and change to meropenem (0.5 g intravenously) every 12 h
2019/8/19	–	Vancomycin (1.0 g intraperitoneally) in an 8-h dwell once at night, stop ceftazidime and change to Amikacin (200 mg intraperitoneally) in a 3-h dwell four times every day	–
2019/8/22	–	Stop Amikacin	–
2019/8/23	–	–	Stop meropenem and change to cefoperazone/sulbactam (1.5 g intravenously) every 12 h
2019/8/26	–	Imipenem/cilastatin (500 mg intraperitoneally) in a 6-h dwell every day	–
2019/8/30	–	–	Stop cefoperazone/sulbactam and change to ampicillin/sulbactam (3 g intravenously) twice every day
2019/9/11	–	Stop imipenem/cilastatin	–
2019/9/16	Discharge and continue amoxicillin (0.25 g orally) three times a day for another 6 days	–	–

The patient and his family were thankful to us and cooperated the further inquiry with us. On further inquiring, the patient reported that he had had a cat at home and when the patient did CAPD, the cat was usually playing with the tubing or contacting the patient during CAPD. However, during his peritoneal dialysis exchange, every step of peritoneal dialysis went all right, without noticing any abnormalities. The patient has suffered from peritoneal dialysis-related peritonitis once in the near 2 years but the causes were not *P. multocida*. He did not report this incident in admission. After treatment, he continues CAPD to do well.

## Discussion and conclusion

**Fig. 2:**
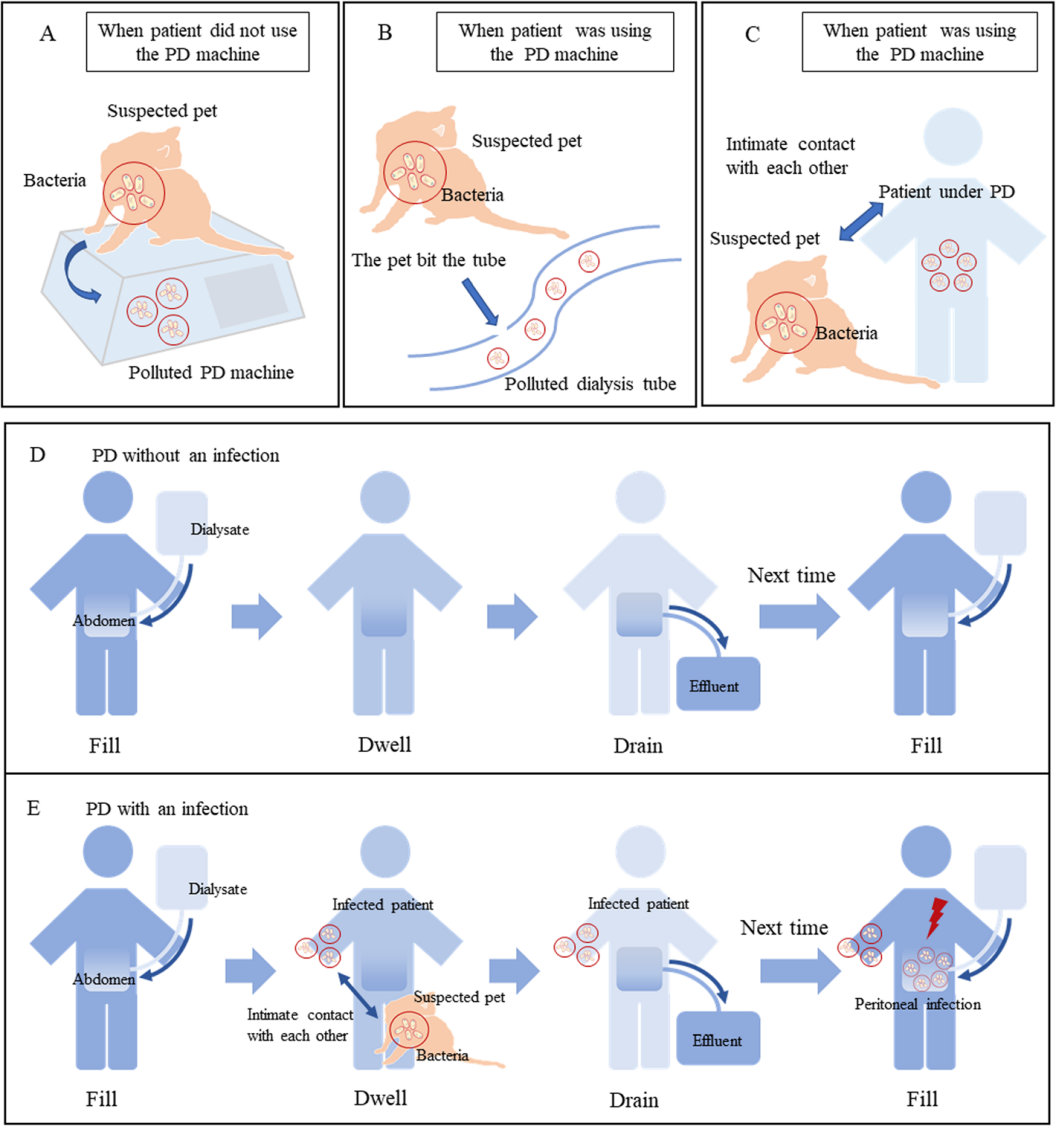
1.Three main risk factors in reviewed cases. **a** When PD machine is not used, the suspected pet plays or rests near the machine transmit the bacteria to the machine. **b** When patient is using the PD machine or is going to use it, the catheter is found to be bit by household pet. **c** When using the PD machine, the patient had intimate contact with the pet. 2.Manual PD approach in this case. **d** The patient was under PD without an infection by suspected pet or other factors. **e** During manual PD, the patient might infect the tube or dialysate after contacting a suspected pet who carried bacteria in process of filling, dwelling and draining. For the next time of PD, the bacteria went into the abdomen and caused peritoneal infection

Patients with ESRD have chosen PD at home to become mainstream. Therefore, the pet-induced peritoneal dialysis-related peritonitis has been taken seriously. *P. multocida* is an animal-sourced Gram-negative coccobacillus, which is generally carried by cats [21]. Except for the usual sites like bit skin and soft tissue, *P. multocida* has been reported to be more high-frequency in patients undergoing PD. Reviewing the peritoneal dialysis-related peritonitis caused by *P. multocida*, we have found three main common occurrences that the patients recalled after *P. multocida* was cultured, which is now considered as risk factors (Fig. 2a, b, c, Table 1). Firstly, when the PD machine was not used, the suspected pet played or rested near the machine, transmit the bacteria to pollute the machine, which in subsequent culture proved to carry bacteria from a pet. Secondly, when a patient was using the PD machine or was going to use it, the catheter was found to be bitten by a household pet, which could be stopped immediately. The last occurrence is when using the PD machine, the patient had intimate contact with the pet. Different from the reviewed case reports, our patient was infected while manual PD without a machine (Fig. 2d, e). During the process of filling, dwelling and draining, the patient might infect the tube and dialysis after contacting the suspected pet carried the bacteria. In the process of manual PD, the patient repeated from filling to draining for at least three times. At the next circulation of the PD process after infected, the bacteria might go into the abdomen and caused the peritoneal infection.

In all cases reviewed, the main clinical complaints were almost similar. The patients complained of a mild-to-severe diffuse abdominal pain with vomiting and nausea usually and the PD effluent was always cloudy or turbid and laboratory tests revealed a high WBC count in PD effluent (Table 1). Clinical features of *P. multocida* induced peritoneal dialysis-related peritonitis are generally not severe but only a few patients have a sensitive reaction, such as low blood pressure. Antibiotics should be initiated into the very beginning of the management of peritonitis, associated with the success of the treatment.

**Fig. 3:**
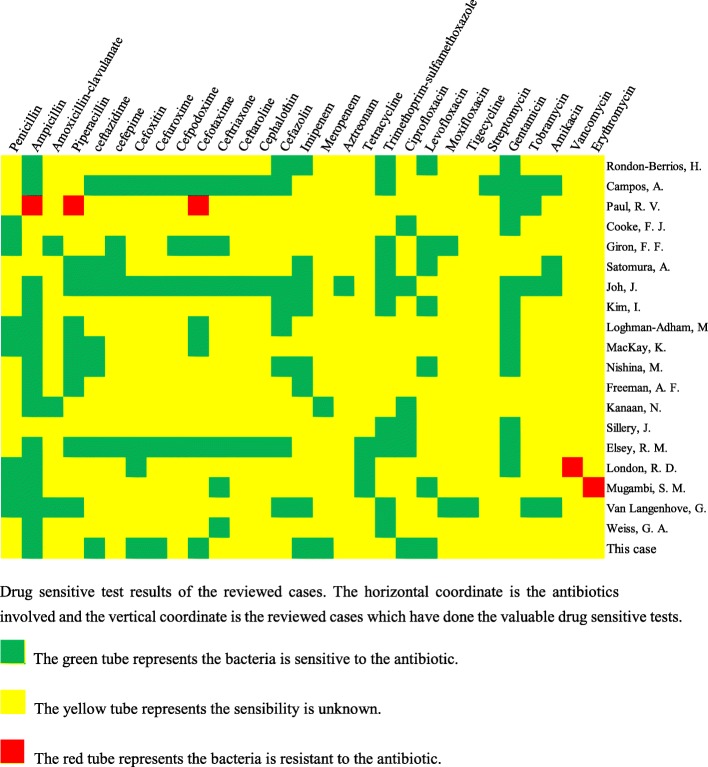
Drug sensitive test results of the reviewed cases. The horizontal coordinate is the antibiotics involved and the vertical coordinate is the reviewed cases which have done the valuable drug sensitive tests.  The green tube represents the bacteria is sensitive to the antibiotic.  The yellow tube represents the sensibility is unknown.  The red tube represents the bacteria is resistant to the antibiotic

[1] The empiric treatments are recommended as a combination of one intraperitoneal antibiotic and one oral or intravenous antibiotic [24]. The antibiotics include penicillin, amoxycillin, fluoroquinolone, the third generation of cephalosporin cefepime, carbon penicillium alkene and compound sulfamethoxazole [25–28]. However, there are no clinical trials specifically demonstrate or evaluate the efficacy of different antibiotics for *P. multocida* related peritonitis. We collected the antibiotic selection and the drug-sensitive test is reviewed case reports and case series studies for the further recommendation (Fig. 3, Table 1). From the collection of each result of drug-sensitive test reviewed, *P. multocida* revealed a low drug resistance and gentamicin, ampicillin and penicillin could be initiated firstly in a selection of the antibiotics. The effectiveness of the recommended antibiotic combination should be detected according to the WBC counts in PD effluent and the antibiotic should be changed if the results are not satisfying. In the management of our patient, firstly we chose meropenem and cefoperazone/sulbactam intravenously and amikacin intraperitoneally with an unsatisfied WBC count in PD effluent. Then we changed treatment by using ampicillin/sulbactam intravenously and the effectiveness was immediate. WBC count in PD effluent is sensitive to the effectiveness of antibiotics and after using ampicillin/sulbactam the WBC count in PD effluent went down and the volume of PD effluent went up (Fig. 1c). Besides, in the use of antibiotics, residual renal function of the patient might be considered which is an extra way of antibiotic excretion [29].

In conclusion, *P. multocida* induced peritoneal dialysis-related peritonitis could be cured by proper antibiotic treatment, but the relapse has not been investigated yet. If individuals keep the pet away from the PD process, the infection route may be severed. It is also important to clean themselves between contact with pets and beginning PD. *P. multocida* induced peritoneal dialysis-related peritonitis does not need catheter removal and exchange with hemodialysis except long-time intractable peritonitis [1, 24, 30]. In our case, considering the family circumstance of the patient, we decided to continue PD with antibiotic treatment beyond the maximum duration recommended by ISPD guideline and finally we, with the patient, overcame the disease. This case is a brave attempt on treatment of those who can not leave from PD or alternate with hemodialysis. However, back to the whole treatment, we still have a deficiency. We have got the result of the drug-sensitive tests early but the follow-up treatment now seems to be a little inexperienced, such as the choice of antibiotic-delivery way and the management of the antibiotics.

## Data Availability

Not applicable.

## Abbreviations

*P. multocida*: *Pasteurella multocida*
ESRD: End stage renal disease
CAPD: Continuous ambulatory peritoneal dialysis
WBC: White blood cell
RBC: Red blood cell
LDH: Lactic dehydrogenase
ADA: Adenosine deaminase
BNP: Natriuretic peptide B
ISPD: International society for prenatal diagnosis
PD: Peritoneal dialysis
HD: Hemodialysis
